# 1-Nonyl-1*H*-benzimidazol-2(3*H*)-one

**DOI:** 10.1107/S1600536810054164

**Published:** 2011-01-08

**Authors:** Younes Ouzidan, Youssef Kandri Rodi, Raymond J. Butcher, El Mokhtar Essassi, Lahcen El Ammari

**Affiliations:** a Laboratoire de Chimie Organique Appliquée, Université Sidi Mohamed Ben Abdallah, Faculté des Sciences et Techniques, Route d’immouzzer, BP 2202 Fès, Morocco; b Department of Chemistry, Howard University, 525 College Street NW, Washington, DC 20059, USA; c INANOTECH (Institute of Nanomaterials and Nanotechnology), MAScIR, Avenue de l’Armée Royale, Rabat, Morocco; d Laboratoire de Chimie du Solide Appliquée, Faculté des Sciences, Université Mohammed V-Agdal, Avenue Ibn Battouta, BP 1014, Rabat, Morocco

## Abstract

The crystal structure of the title compound, C_16_H_24_N_2_O, is built up from two fused six- and five-membered rings linked to C_9_H_19_ chains. The fused-ring system is essentially planar, the largest deviation from the mean plane being 0.009 (2) Å. The chain is nearly perpendicular to this plane [dihedral angle = 80.27 (17)°]. In the crystal, inter­molecular N—H⋯O hydrogen bonds form dimers with an *R*_2_^2^(8) graph-set motif. These dimers are further connected through C—H⋯O hydrogen bonds, building sheets parallel to (100).

## Related literature

For the pharmacological and biochemical properties of benzimidazol-2-one derivatives, see: El Azzaoui *et al.* (2006 ▶); Soderlind *et al.* (1999 ▶); Rémond *et al.* (1997 ▶); Gribkoff *et al.* (1994 ▶); Olesen *et al.* (1994 ▶); McKay *et al.* (1994 ▶). For hydrogen-bond motifs, see: Etter *et al.* (1990 ▶); Bernstein *et al.* (1995 ▶). 
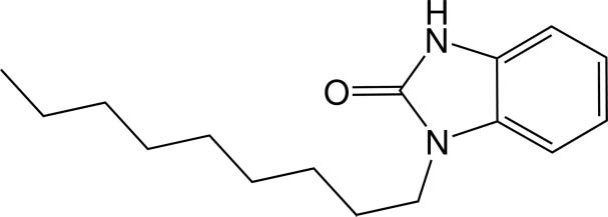


## Experimental

### 

#### Crystal data

C_16_H_24_N_2_O
*M* *_r_* = 260.37
Monoclinic, 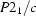

*a* = 18.023 (1) Å
*b* = 5.4585 (2) Å
*c* = 16.5708 (9) Å
β = 115.543 (7)°
*V* = 1470.86 (15) Å^3^
*Z* = 4
Cu *K*α radiation
μ = 0.57 mm^−1^
*T* = 123 K
0.54 × 0.14 × 0.08 mm

#### Data collection

Oxford Diffraction Xcalibur Ruby Gemini diffractometer
Absorption correction: multi-scan (*CrysAlis PRO*; Oxford Diffraction, 2010 ▶) *T*_min_ = 0.908, *T*_max_ = 0.955
4966 measured reflections
2656 independent reflections
2073 reflections with *I* > 2σ(*I*)
*R*_int_ = 0.038

#### Refinement

*R*[*F* ^2^ > 2σ(*F* ^2^)] = 0.048
*wR*(*F* ^2^) = 0.137
*S* = 1.06
2656 reflections
177 parameters
H atoms treated by a mixture of independent and constrained refinement
Δρ_max_ = 0.24 e Å^−3^
Δρ_min_ = −0.23 e Å^−3^

### 

Data collection: *CrysAlis PRO* (Oxford Diffraction, 2010 ▶); cell refinement: *CrysAlis PRO*; data reduction: *CrysAlis PRO*; program(s) used to solve structure: *SHELXS97* (Sheldrick, 2008 ▶); program(s) used to refine structure: *SHELXL97* (Sheldrick, 2008 ▶); molecular graphics: *ORTEP-3 for Windows* (Farrugia, 1997 ▶); software used to prepare material for publication: *WinGX* (Farrugia, 1999 ▶).

## Supplementary Material

Crystal structure: contains datablocks I, global. DOI: 10.1107/S1600536810054164/dn2641sup1.cif

Structure factors: contains datablocks I. DOI: 10.1107/S1600536810054164/dn2641Isup2.hkl

Additional supplementary materials:  crystallographic information; 3D view; checkCIF report

## Figures and Tables

**Table 1:** Hydrogen-bond geometry (Å, °)

*D*—H⋯*A*	*D*—H	H⋯*A*	*D*⋯*A*	*D*—H⋯*A*
N2—H2*N*⋯O1^i^	0.92 (2)	1.92 (2)	2.817 (2)	166.1 (19)
C4—H4*A*⋯O1^ii^	0.95	2.50	3.284 (2)	140
C8—H8*B*⋯O1^iii^	0.99	2.55	3.453 (2)	151

Symmetry codes: (i) 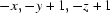; (ii) 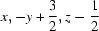; (iii) 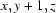.

**Fig. 1.**
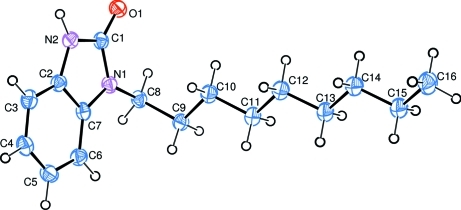
Molecular structure of the title compound with the atom-labelling scheme. Displacement ellipsoids are drawn at the 50% probability level. H atoms are represented as small circles.

## supplementary crystallographic information

### Comment

Benzimidazol-2-one derivatives are useful heterocyclic building blocks (El Azzaoui *et al.*,2006) and are prominent structural elements of compounds demonstrating a wide variety of pharmacological and biochemical properties (Soderlind *et al.*,1999). Examples of pharmacological activity exhibited by benzimidazol-2-ones include antagonism of neurotransmitter receptors, inhibition of aldose reductase, antiulcer and antisecretory properties, and modulation of ion channels. (Rémond *et al.*, (1997); Gribkoff *et al.*, (1994); Olesen *et al.*, (1994); McKay *et al.*, (1994).

The 1-nonyl-1*H*-benzimidazol-2(3*H*)-one molecule structure is built up from two fused six-and five-membered rings linked to C_9_H_19_ chains as schown in Fg.1. The fused-ring system is essentially planar, with a maximum deviation of 0.005 (2) Å and -0.009 (2) Å for C7 and N1 respectyvely. The dihedral angle between them does not exceed 1.03 (6)°. The torsion angles C1 N1 C8 C9 and C11 C12 C13 C14 are 113.4 (2)° and 178.9 (2)° respectively.

N-H···O hydrogen bonds result in the formation of dimers with R_2_^2^(8) graph set motif (Etter *et al.*, 1990; Bernstein *et al.*, 1995). These dimer are further connected through C-H···O hydrogen bonds building sheets parallell to the (1 0 0) plane. (Table 1).

### Experimental

To benzimidazol-2-one (0,21 g, 1,5 mmol), potassium carbonate (0,41 g, 3 mmol), and tetra-n-butylammonium bromide (0.1 g, 0,3 mmol) in DMF (15 ml) was added 1-bromononane (0,57 ml, 3 mmol). Stirring was continued at room temperature for 6 h. The salts were removed by filtration and the filtrate concentrated under reduced pressure. The residue was separated by chromatography on a column of silica gel with ethyl acetate/hexane (1/2) as eluent. Colorless crystals were isolated when the solvent was allowed to evaporate.

### Refinement

H atoms were located in a difference map and treated as riding with C—H = 0.93 Å for all H atoms with *U*_iso_(H) = 1.2 *U*_eq_(aromatic, methine)and *U*_iso_(H) = 1.5 *U*_eq_(methyl).

### Crystal data

**Table:**

C_16_H_24_N_2_O	*F*(000) = 568
*M**_r_* = 260.37	*D*_x_ = 1.176 Mg m^−^^3^
Monoclinic, *P*2_1_/*c*	Cu *K*α radiation, λ = 1.54184 Å
Hall symbol: -P 2ybc	Cell parameters from 2656 reflections
*a* = 18.023 (1) Å	θ = 5.3–67.7°
*b* = 5.4585 (2) Å	µ = 0.57 mm^−^^1^
*c* = 16.5708 (9) Å	*T* = 123 K
β = 115.543 (7)°	Needle, colorless
*V* = 1470.86 (15) Å^3^	0.54 × 0.14 × 0.08 mm
*Z* = 4	

### Data collection

**Table:**

Oxford Diffraction Xcalibur Ruby Gemini diffractometer	2656 independent reflections
Radiation source: Enhance (Cu) X-ray Source	2073 reflections with *I* > 2σ(*I*)
graphite	*R*_int_ = 0.038
Detector resolution: 10.5081 pixels mm^-1^	θ_max_ = 67.7°, θ_min_ = 5.3°
ω scans	*h* = −21→14
Absorption correction: multi-scan (*CrysAlis PRO*; Oxford Diffraction, 2010)	*k* = −6→5
*T*_min_ = 0.908, *T*_max_ = 0.955	*l* = −15→19
4966 measured reflections	

### Refinement

**Table:**

Refinement on *F*^2^	Primary atom site location: structure-invariant direct methods
Least-squares matrix: full	Secondary atom site location: difference Fourier map
*R*[*F*^2^ > 2σ(*F*^2^)] = 0.048	Hydrogen site location: inferred from neighbouring sites
*wR*(*F*^2^) = 0.137	H atoms treated by a mixture of independent and constrained refinement
*S* = 1.06	*w* = 1/[σ^2^(*F*_o_^2^) + (0.0758*P*)^2^ + 0.0676*P*] where *P* = (*F*_o_^2^ + 2*F*_c_^2^)/3
2656 reflections	(Δ/σ)_max_ < 0.001
177 parameters	Δρ_max_ = 0.24 e Å^−^^3^
0 restraints	Δρ_min_ = −0.23 e Å^−^^3^

### Special details

**Table:**

Experimental. CrysAlisPro, Oxford Diffraction Ltd (2010). Version 1.171.34.36 (release 02-08-2010 CrysAlis171 .NET). Empirical absorption correction using spherical harmonics, implemented in SCALE3 ABSPACK scaling algorithm.
Geometry. All e.s.d.'s (except the e.s.d. in the dihedral angle between two l.s. planes) are estimated using the full covariance matrix. The cell e.s.d.'s are taken into account individually in the estimation of e.s.d.'s in distances, angles and torsion angles; correlations between e.s.d.'s in cell parameters are only used when they are defined by crystal symmetry. An approximate (isotropic) treatment of cell e.s.d.'s is used for estimating e.s.d.'s involving l.s. planes.
Refinement. Refinement of *F*^2^ against ALL reflections. The weighted *R*-factor *wR* and goodness of fit *S* are based on *F*^2^, conventional *R*-factors *R* are based on *F*, with *F* set to zero for negative *F*^2^. The threshold expression of *F*^2^ > 2σ(*F*^2^) is used only for calculating *R*-factors(gt) *etc*. and is not relevant to the choice of reflections for refinement. *R*-factors based on *F*^2^ are statistically about twice as large as those based on *F*, and *R*- factors based on ALL data will be even larger.

### Fractional atomic coordinates and isotropic or equivalent isotropic displacement parameters (Å^2^)

**Table:**

	*x*	*y*	*z*	*U*_iso_*/*U*_eq_	
O1	0.04944 (8)	0.7205 (2)	0.58716 (8)	0.0258 (3)	
N1	0.11884 (9)	0.9981 (3)	0.53791 (9)	0.0227 (3)	
N2	0.05350 (9)	0.6926 (3)	0.44838 (9)	0.0238 (3)	
H2N	0.0218 (14)	0.554 (4)	0.4288 (14)	0.040 (6)*	
C1	0.07126 (10)	0.7949 (3)	0.53033 (11)	0.0217 (4)	
C2	0.08761 (11)	0.8328 (3)	0.40292 (11)	0.0231 (4)	
C3	0.08572 (11)	0.8103 (3)	0.31892 (12)	0.0269 (4)	
H3A	0.0576	0.6787	0.2803	0.032*	
C4	0.12671 (11)	0.9882 (3)	0.29310 (12)	0.0285 (4)	
H4A	0.1271	0.9765	0.2361	0.034*	
C5	0.16711 (12)	1.1829 (3)	0.34929 (12)	0.0288 (4)	
H5A	0.1941	1.3020	0.3296	0.035*	
C6	0.16872 (11)	1.2065 (3)	0.43378 (12)	0.0256 (4)	
H6A	0.1960	1.3396	0.4720	0.031*	
C7	0.12889 (10)	1.0278 (3)	0.45958 (11)	0.0228 (4)	
C8	0.14782 (11)	1.1628 (3)	0.61482 (11)	0.0237 (4)	
H8A	0.1212	1.1168	0.6539	0.028*	
H8B	0.1306	1.3320	0.5935	0.028*	
C9	0.24066 (11)	1.1583 (3)	0.66960 (11)	0.0244 (4)	
H9A	0.2555	1.2741	0.7202	0.029*	
H9B	0.2670	1.2171	0.6317	0.029*	
C10	0.27540 (11)	0.9070 (3)	0.70653 (12)	0.0282 (4)	
H10A	0.2660	0.7946	0.6561	0.034*	
H10B	0.2457	0.8404	0.7399	0.034*	
C11	0.36729 (11)	0.9157 (4)	0.76838 (12)	0.0287 (4)	
H11A	0.3963	0.9927	0.7360	0.034*	
H11B	0.3761	1.0207	0.8204	0.034*	
C12	0.40510 (11)	0.6654 (4)	0.80204 (12)	0.0304 (4)	
H12A	0.3996	0.5635	0.7503	0.036*	
H12B	0.3741	0.5839	0.8314	0.036*	
C13	0.49577 (12)	0.6790 (4)	0.86815 (12)	0.0300 (4)	
H13A	0.5267	0.7583	0.8383	0.036*	
H13B	0.5012	0.7841	0.9191	0.036*	
C14	0.53471 (11)	0.4315 (3)	0.90421 (12)	0.0301 (4)	
H14A	0.5305	0.3269	0.8536	0.036*	
H14B	0.5035	0.3506	0.9335	0.036*	
C15	0.62484 (11)	0.4504 (4)	0.97121 (12)	0.0314 (4)	
H15A	0.6556	0.5360	0.9425	0.038*	
H15B	0.6288	0.5511	1.0226	0.038*	
C16	0.66513 (13)	0.2034 (4)	1.00569 (14)	0.0392 (5)	
H16A	0.7224	0.2283	1.0491	0.059*	
H16B	0.6637	0.1050	0.9556	0.059*	
H16C	0.6353	0.1176	1.0346	0.059*	

### Atomic displacement parameters (Å^2^)

**Table:**

	*U*^11^	*U*^22^	*U*^33^	*U*^12^	*U*^13^	*U*^23^
O1	0.0284 (7)	0.0254 (7)	0.0233 (6)	−0.0011 (5)	0.0109 (5)	0.0012 (5)
N1	0.0246 (8)	0.0227 (8)	0.0190 (7)	0.0001 (6)	0.0077 (6)	0.0002 (6)
N2	0.0242 (8)	0.0228 (8)	0.0225 (8)	−0.0017 (6)	0.0083 (6)	−0.0009 (6)
C1	0.0202 (8)	0.0209 (8)	0.0214 (8)	0.0037 (7)	0.0066 (7)	0.0018 (7)
C2	0.0203 (8)	0.0227 (9)	0.0241 (9)	0.0019 (7)	0.0075 (7)	0.0012 (7)
C3	0.0265 (9)	0.0269 (10)	0.0231 (9)	0.0010 (7)	0.0065 (7)	−0.0024 (7)
C4	0.0306 (10)	0.0331 (10)	0.0216 (8)	0.0037 (8)	0.0112 (8)	0.0025 (8)
C5	0.0301 (10)	0.0290 (10)	0.0283 (10)	0.0003 (8)	0.0135 (8)	0.0044 (7)
C6	0.0254 (9)	0.0227 (9)	0.0258 (9)	−0.0010 (7)	0.0082 (7)	−0.0005 (7)
C7	0.0211 (9)	0.0246 (9)	0.0202 (8)	0.0048 (7)	0.0065 (7)	0.0026 (7)
C8	0.0266 (9)	0.0224 (9)	0.0203 (8)	0.0009 (7)	0.0083 (7)	−0.0006 (7)
C9	0.0257 (9)	0.0246 (9)	0.0218 (9)	−0.0014 (7)	0.0093 (7)	−0.0015 (7)
C10	0.0258 (10)	0.0274 (10)	0.0272 (9)	−0.0001 (8)	0.0076 (8)	0.0010 (7)
C11	0.0253 (10)	0.0310 (10)	0.0254 (9)	−0.0003 (8)	0.0066 (8)	0.0017 (7)
C12	0.0274 (10)	0.0312 (10)	0.0281 (9)	−0.0006 (8)	0.0077 (8)	0.0008 (8)
C13	0.0267 (10)	0.0304 (10)	0.0279 (10)	0.0013 (8)	0.0071 (8)	0.0026 (8)
C14	0.0272 (10)	0.0307 (10)	0.0293 (9)	0.0007 (8)	0.0092 (8)	0.0022 (8)
C15	0.0278 (10)	0.0320 (10)	0.0299 (10)	0.0015 (8)	0.0080 (8)	0.0030 (8)
C16	0.0313 (11)	0.0384 (12)	0.0405 (12)	0.0061 (9)	0.0084 (9)	0.0048 (9)

### Geometric parameters (Å, °)

**Table:**

O1—C1	1.235 (2)	C9—H9B	0.9900
N1—C1	1.375 (2)	C10—C11	1.527 (2)
N1—C7	1.395 (2)	C10—H10A	0.9900
N1—C8	1.460 (2)	C10—H10B	0.9900
N2—C1	1.372 (2)	C11—C12	1.522 (3)
N2—C2	1.389 (2)	C11—H11A	0.9900
N2—H2N	0.92 (2)	C11—H11B	0.9900
C2—C3	1.383 (2)	C12—C13	1.527 (3)
C2—C7	1.402 (2)	C12—H12A	0.9900
C3—C4	1.395 (3)	C12—H12B	0.9900
C3—H3A	0.9500	C13—C14	1.521 (3)
C4—C5	1.393 (3)	C13—H13A	0.9900
C4—H4A	0.9500	C13—H13B	0.9900
C5—C6	1.394 (2)	C14—C15	1.525 (2)
C5—H5A	0.9500	C14—H14A	0.9900
C6—C7	1.384 (2)	C14—H14B	0.9900
C6—H6A	0.9500	C15—C16	1.521 (3)
C8—C9	1.521 (2)	C15—H15A	0.9900
C8—H8A	0.9900	C15—H15B	0.9900
C8—H8B	0.9900	C16—H16A	0.9800
C9—C10	1.523 (2)	C16—H16B	0.9800
C9—H9A	0.9900	C16—H16C	0.9800
			
C1—N1—C7	109.57 (14)	C11—C10—H10A	109.1
C1—N1—C8	123.43 (14)	C9—C10—H10B	109.1
C7—N1—C8	126.84 (15)	C11—C10—H10B	109.1
C1—N2—C2	110.10 (15)	H10A—C10—H10B	107.9
C1—N2—H2N	121.8 (13)	C12—C11—C10	113.73 (16)
C2—N2—H2N	128.0 (13)	C12—C11—H11A	108.8
O1—C1—N2	127.30 (17)	C10—C11—H11A	108.8
O1—C1—N1	125.93 (16)	C12—C11—H11B	108.8
N2—C1—N1	106.77 (14)	C10—C11—H11B	108.8
C3—C2—N2	132.22 (17)	H11A—C11—H11B	107.7
C3—C2—C7	121.15 (16)	C11—C12—C13	113.00 (16)
N2—C2—C7	106.63 (15)	C11—C12—H12A	109.0
C2—C3—C4	117.41 (17)	C13—C12—H12A	109.0
C2—C3—H3A	121.3	C11—C12—H12B	109.0
C4—C3—H3A	121.3	C13—C12—H12B	109.0
C5—C4—C3	121.29 (16)	H12A—C12—H12B	107.8
C5—C4—H4A	119.4	C14—C13—C12	114.10 (16)
C3—C4—H4A	119.4	C14—C13—H13A	108.7
C4—C5—C6	121.35 (17)	C12—C13—H13A	108.7
C4—C5—H5A	119.3	C14—C13—H13B	108.7
C6—C5—H5A	119.3	C12—C13—H13B	108.7
C7—C6—C5	117.15 (17)	H13A—C13—H13B	107.6
C7—C6—H6A	121.4	C13—C14—C15	113.07 (16)
C5—C6—H6A	121.4	C13—C14—H14A	109.0
C6—C7—N1	131.46 (16)	C15—C14—H14A	109.0
C6—C7—C2	121.64 (16)	C13—C14—H14B	109.0
N1—C7—C2	106.89 (15)	C15—C14—H14B	109.0
N1—C8—C9	113.48 (14)	H14A—C14—H14B	107.8
N1—C8—H8A	108.9	C16—C15—C14	113.53 (17)
C9—C8—H8A	108.9	C16—C15—H15A	108.9
N1—C8—H8B	108.9	C14—C15—H15A	108.9
C9—C8—H8B	108.9	C16—C15—H15B	108.9
H8A—C8—H8B	107.7	C14—C15—H15B	108.9
C8—C9—C10	114.21 (15)	H15A—C15—H15B	107.7
C8—C9—H9A	108.7	C15—C16—H16A	109.5
C10—C9—H9A	108.7	C15—C16—H16B	109.5
C8—C9—H9B	108.7	H16A—C16—H16B	109.5
C10—C9—H9B	108.7	C15—C16—H16C	109.5
H9A—C9—H9B	107.6	H16A—C16—H16C	109.5
C9—C10—C11	112.36 (16)	H16B—C16—H16C	109.5
C9—C10—H10A	109.1		
			
C2—N2—C1—O1	178.67 (16)	C8—N1—C7—C6	2.0 (3)
C2—N2—C1—N1	−1.54 (18)	C1—N1—C7—C2	−1.36 (18)
C7—N1—C1—O1	−178.42 (16)	C8—N1—C7—C2	−176.97 (15)
C8—N1—C1—O1	−2.6 (3)	C3—C2—C7—C6	0.6 (3)
C7—N1—C1—N2	1.78 (18)	N2—C2—C7—C6	−178.71 (16)
C8—N1—C1—N2	177.57 (14)	C3—C2—C7—N1	179.70 (15)
C1—N2—C2—C3	−178.49 (18)	N2—C2—C7—N1	0.39 (18)
C1—N2—C2—C7	0.71 (19)	C1—N1—C8—C9	113.44 (17)
N2—C2—C3—C4	179.37 (18)	C7—N1—C8—C9	−71.5 (2)
C7—C2—C3—C4	0.3 (2)	N1—C8—C9—C10	−58.51 (19)
C2—C3—C4—C5	−0.8 (3)	C8—C9—C10—C11	−174.43 (14)
C3—C4—C5—C6	0.5 (3)	C9—C10—C11—C12	−176.51 (15)
C4—C5—C6—C7	0.3 (3)	C10—C11—C12—C13	−176.55 (14)
C5—C6—C7—N1	−179.73 (17)	C11—C12—C13—C14	178.92 (15)
C5—C6—C7—C2	−0.9 (3)	C12—C13—C14—C15	−179.06 (15)
C1—N1—C7—C6	177.62 (18)	C13—C14—C15—C16	−178.32 (16)

### Hydrogen-bond geometry (Å, °)

**Table:**

*D*—H···*A*	*D*—H	H···*A*	*D*···*A*	*D*—H···*A*
N2—H2N···O1^i^	0.92 (2)	1.92 (2)	2.817 (2)	166.1 (19)
C4—H4A···O1^ii^	0.95	2.50	3.284 (2)	140
C8—H8B···O1^iii^	0.99	2.55	3.453 (2)	151

Symmetry codes: (i) −*x*, −*y*+1, −*z*+1; (ii) *x*, −*y*+3/2, *z*−1/2; (iii) *x*, *y*+1, *z*.
